# Discrimination of Maturity Stages of Cabernet Sauvignon Wine Grapes Using Visible–Near-Infrared Spectroscopy

**DOI:** 10.3390/foods12234371

**Published:** 2023-12-04

**Authors:** Xuejian Zhou, Wenzheng Liu, Kai Li, Dongqing Lu, Yuan Su, Yanlun Ju, Yulin Fang, Jihong Yang

**Affiliations:** 1 College of Enology, Northwest A&F University, Yangling 712100, China; zhouxuejian@nwafu.edu.cn (X.Z.); lwzheng@nwafu.edu.cn (W.L.); likai@nwafu.edu.cn (K.L.); ludongqing@nwafu.edu.cn (D.L.); suyuan@nwafu.edu.cn (Y.S.); juyanlun2016@nwsuaf.edu.cn (Y.J.); fangyulin@nwsuaf.edu.cn (Y.F.); 2 College of Food Science and Pharmacy, Xinjiang Agricultural University, Urumqi 830052, China; 3 Shaanxi Engineering Research Center for Viti-Viniculture, Yangling 712100, China

**Keywords:** Vis-NIR spectroscopy, wine grape, 1D-CNN, SAE, ripeness

## Abstract

Grape quality and ripeness play a crucial role in producing exceptional wines with high-value characteristics, which requires an effective assessment of grape ripeness. The primary purpose of this research is to explore the possible application of visible–near-infrared spectral (Vis-NIR) technology for classifying the maturity stages of wine grapes based on quality indicators. The reflection spectra of Cabernet Sauvignon grapes were recorded using a spectrometer in the spectral range of 400 nm to 1029 nm. After measuring the soluble solids content (SSC), total acids (TA), total phenols (TP), and tannins (TN), the grape samples were categorized into five maturity stages using a spectral clustering method. A traditional supervised classification method, a support vector machine (SVM), and two deep learning techniques, namely stacked autoencoders (SAE) and one-dimensional convolutional neural networks (1D-CNN), were employed to construct a discriminant model and investigate the association linking grape maturity stages and the spectral responses. The spectral data went through three commonly used preprocessing methods, and feature wavelengths were extracted using a competitive adaptive reweighting algorithm (CARS). The spectral data model preprocessed via multiplicative scattering correction (MSC) outperformed the other two preprocessing methods. After preprocessing, a comparison was made between the discriminant models established with full and effective spectral data. It was observed that the SAE model, utilizing the feature spectrum, demonstrated superior overall performance. The classification accuracies of the calibration and prediction sets were 100% and 94%, respectively. This study showcased the dependability of combining Vis-NIR spectroscopy with deep learning methods for rapidly and accurately distinguishing the ripeness stage of grapes. It has significant implications for future applications in wine production and the development of optoelectronic instruments tailored to the specific needs of the winemaking industry.

## 1. Introduction

Grape (*Vitis vinifera* L.), an esteemed fruit crop, holds a prominent position within the global agricultural landscape. Its versatile applications encompass consumption as fresh fruits (fresh grapes), dried fruits (raisins), juice, and the majority of grapes serve as the cornerstone of wine production [1]. A growing consumer emphasis on quality has exerted a profound influence on the vinification process [2]. The maturation stage of wine grapes is of paramount importance in determining the resultant wine’s quality and significantly influences the timing of the grape harvest [3]. Distinct types of wines necessitate specific degrees of grape ripeness. If the grapes are underripe, they fail to meet the requisite physical and chemical parameters essential for brewing, which results in wines with heightened bitterness and astringency. Conversely, excessive ripeness can lead to fruit softening and challenging postharvest storage. Furthermore, flavors diminish rapidly, and overripe aromas may pervade the resulting wine [4]. Research [5] has indicated that several factors profoundly impact grape maturity and wine quality, including soluble solids content (SSC), total acids (TA), and phenolic substances. The SSC directly influences the alcohol content of the wine, while grape quality and flavor, as well as the wine’s structure and freshness, vitally depend on the TA. Phenolic compounds assume significance in determining the wine’s color, flavor, and taste, underscoring the essentiality of monitoring such compounds during the maturation process. Nevertheless, the conventional approach to measuring these parameters necessitates the physical disruption of a grape bunch, rendering it impractical and resource-intensive. This traditional method demands a substantial number of samples, contributing to significant time delays and economic inefficiencies. Moreover, it mandates the involvement of skilled operators possessing specialized knowledge in the field [6].

Recently, the advent of chemometrics and advancements in instrumentation have paved the way for the application of spectroscopic techniques in assessing the internal quality of fruits and vegetables. Spectral technologies, encompassing visible light, near-infrared, mid-infrared, and hyperspectral spectroscopy, have found widespread utility in the quest for gauging the quality of grapes. By examining the spectral profiles of grapes, one can extract data pertaining to various quality indicators [7,8]. This approach offers the notable advantages of simplicity and rapidity compared to traditional physical and chemical measurement methods. Consequently, numerous studies have delved into employing spectral detection methods for analyzing fruit maturity. Li et al. [9] conducted a study that explored the viability of employing a multi-cultivar model to accurately determine the SSC in three pear cultivars, utilizing visible-NIR spectroscopy in the range of 350–1800 nm. The results obtained from their investigation were notably satisfactory. Zhang et al. [10] employed a hyperspectral imaging system that encompassed two distinct spectral ranges (380–1030 nm and 874–1734 nm) to assess the ripeness of strawberries at three different stages of maturation. Their research revealed that the utilization of datasets obtained from hyperspectral images within the range of 441.1–1013.97 nm resulted in better classification accuracy that yielded meaningful insights into the evaluation of strawberry ripeness. Fernández-Novales et al. [11] assessed the grape amino acid content throughout the maturation process using both visible (570–1000 nm) and near-infrared (1100–2100 nm) spectroscopy on intact grape berries. By investigating these spectral ranges, they gained valuable results regarding an estimation of the amino acid content in grapes as they ripen. Xiao et al. [12] conducted a comprehensive study on the application of visible-near infrared (vis/NIR) spectroscopy spanning from 400 nm to 1100 nm for the classification of grape berries based on their SSC and total phenol (TP) quality parameters. By implementing a partial least squares discrimination analysis (PLS-DA), they successfully utilized the vis/NIR spectrum to differentiate between grape berries with varying SSC and TP concentrations, achieving an accuracy of over 77%. These studies collectively highlight the remarkable potential of Vis-NIR or NIR spectroscopy for assessing the maturity of diverse fruits. Nevertheless, our literature search indicated a scarcity of reports specifically focusing on wine grapes and the utilization of Vis-NIR spectroscopy for classifying ripening stages based on internal quality indicators. Furthermore, in previous studies, the challenge of achieving handheld and portable operations for determining fruit ripeness has been prominent, often necessitating the use of laboratory instruments.

Vis-NIR spectra are often characterized by their complexity and noisy nature, making traditional machine learning methods a proven choice for processing such data. In this study, two distinct approaches are employed to recognize spectral features associated with grape ripening stages. First, support vector machine (SVM), a traditional multivariate supervised classification method, is utilized. Additionally, deep learning methods have gained significant attention in the field of artificial intelligence, representing a wide range of machine learning algorithms that possess formidable pattern recognition capabilities that render them highly suitable for extracting valuable information from extensive spectral databases. In such databases, inherent nonlinearity arises from intricate biological, environmental, and instrumental variations [13]. Tsakiridis et al. [14] implemented two different autoencoder architectures to transform the original recorded spectra into standardized reflectance spectra without considering illumination conditions. Their study confirmed the suitability of the convolutional autoencoder framework. Silva et al. [15] applied two state-of-the-art (SOTA) convolutional networks, namely InceptionTime and OmniScale 1D-CNN, to predict the sugar content of winemaking grape berries using hyperspectral images. Both models demonstrated excellent generalization ability and produced highly competitive results across different grape varieties and vintages. Zheng et al. [16] compared traditional partial least squares regression (PLSR) with 1D-CNN modeling methods under the influence of two external factors. The results indicated that 1D-CNN can serve as a more convenient alternative for online determination of soluble solids content (SSC) in apples, significantly reducing the complexity of the Vis/NIR spectral modeling process. Some studies have also applied deep learning methods for the qualitative discrimination of fruits, yielding commendable results [17,18]. However, most of these studies only focused on a few individual indicators, such as sugars and acids. In this article, we employed multiple indicators to determine the maturity stages, including sugars, acids, and phenolic compounds. Furthermore, two deep learning methods, namely stacked autoencoder (SAE) and one-dimensional convolutional neural network (1D-CNN), were utilized for the classification of maturity stages, and their predictive capabilities were compared to those of traditional supervised classification methods.

Specifically, this study focuses on accomplishing the following key objectives: (1) Constructing discriminant models, namely SVM, 1D-CNN, and SAE models, for the entire spectrum preprocessed using the multiple scattering correction (MSC), standard normative variate (SNV), and Savitzky–Golay smoothing (S-G smoothing) techniques. These models will subsequently be compared to determine their effectiveness. (2) Employing the competitive adaptive reweighted sampling (CARS) method to extract the most significant spectral wavelengths that are crucial for accurate discrimination. (3) Developing a streamlined discriminant model utilizing the selected spectral wavelengths and evaluating its performance against the model based on the full spectrum. Ultimately, the discriminant model exhibiting the highest classification accuracy will be identified and recommended for adoption.

## 2. Materials and Methods

### 2.1. Sample Collection

**Figure 1:**
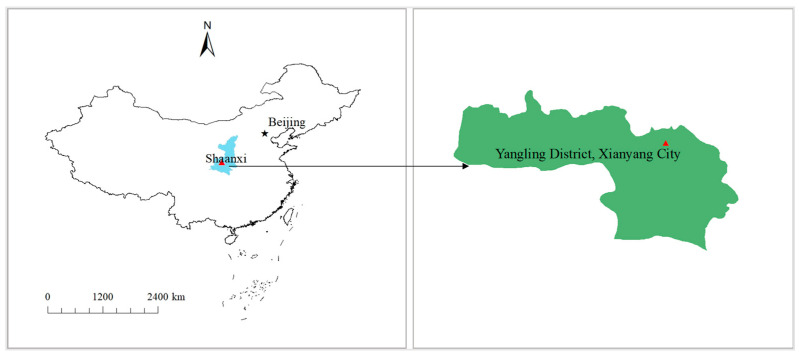
Map of grape collection location.

Grape samples (*Vitis vinifera* L. cv. Cabernet Sauvignon) were meticulously harvested by seasoned growers from Cao Xinzhuang vineyard (34°18′0″ N; 108°5′23.9″ E, Yangling, China) (Figure 1) over the months of July and August 2022. To ensure adequate variability, the stratified sampling method was adopted. Two rows of plants were designated as sampling locations, and various grape clusters were meticulously chosen from different parts of each plant [19]. The selected grape clusters were divided into three positions: top, middle, and bottom. From each position, two berries were handpicked, resulting in a total of six berries per grape cluster. These samples were considered to represent the entirety of the grape cluster. Samples were collected weekly during veraison and then every three to four days until commercial harvest. On each sampling day, 300 grapes were collected between 7:00 and 9:00 am. Furthermore, to enhance the model’s accuracy, the grape samples were divided into 10 groups based on their similar physiological status [20].

### 2.2. Vis-NIR Reflectance Spectral Data Obtainment

**Figure 2:**
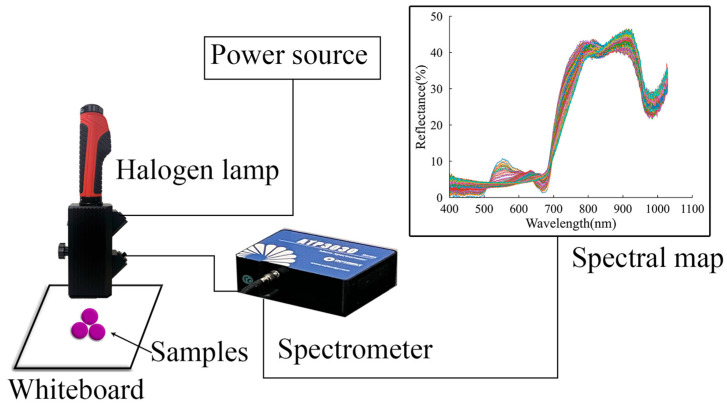
Reflectance spectral acquisition system.

A diffuse reflectance spectral analysis was used to acquire the spectrum in this experiment. To acquire spectral data, an ultra-high-resolution fiber-optic spectral system was used that consists of the following components: a high-resolution micro-spectrometer ATP3030 (Optosky Photonics Inc., Xiamen, China) with a built-in circuit that can synchronously trigger and control a xenon lamp, operating with a wavelength range of 200 nm up to 1100 nm and a resolution from 0.05 nm to 2 nm; a 12 V bulb halogen light source/HL2000 (Optosky Photonics Inc., China); a dark chamber; optical fibers; and a computer with Optosky Spectra V3.1.25 software (Optosky Photonics Inc., Xiamen, China). A standard whiteboard (Guangzhou Jingyi Optoelectronic Technology Inc., Xiamen, China) was used as a white reference, with which calibration operations (standard white reference definition) were implemented before data acquisition. The grapes were placed on a whiteboard for spectral acquisition, as shown in Figure 2. Each sample (30 grapes) included 10 subsamples, and each subsample consisted of three grapes. The regions between the 200 nm to 400 nm and 1030 nm to 1100 nm bands were removed from the analysis due to excessive random noise. In addition, the Euclidean distance was utilized to analyze the multiple spectral data collected for each sample, enabling the identification of the spectral data with the minimum distance to other spectral data within the same sample [21]. These selected spectral data were then used for modeling.

### 2.3. Measurement of the Physicochemical Parameters

#### 2.3.1. Grape Pretreatment

After the reflectance measurements, the individual samples were frozen at −40 °C for the reference measurements until the manual separation of skin, pulp, and seeds. Subsequently, the pulp was made into grape juice for quantifying SSC and TA. The skin fraction was frozen for the total phenol and tannin analyses.

Prior to the phenolic substance analysis, the skin was subjected to a process of extracting the solution preparation. Before preparing the solution, the skin was crushed into a powder under freezing conditions using liquid nitrogen. The powder was placed into a vacuum freeze dryer (Shanghai Yetuo Technology Co., Ltd., Shanghai, China) for 24 h. Each sample was then macerated in the hydroalcoholic acid solution (HCL 0.1% *v*/*v*: methanol 60% *v*/*v*) at 30 °C, 40 power for 30 min under ultrasonication. The mixture was then centrifuged at 13,710× *g* for 10 min, and the supernatant was collected. The above process was repeated twice, and the supernatants were stored in a refrigerator at −40 °C for the measurement of phenolic substances.

#### 2.3.2. SSC and TA Determination

As a reference, the SSC and TA values of the samples were analyzed using standard destructive methods. SSC measurements of the juice were conducted from the direct reading in a handheld digital brix refractometer (Guangzhou Ai Measure Intelligent Technology Co., Ltd., Guangzhou, China) and were expressed in °Brix with an accuracy of 0.1 °Brix. A Mettler automatic potentiometric titrator (Mettler-Toledo International Inc., Zurich, Switzerland) was applied to measure the TA content. The above process was conducted in triplicate. The SSC/TA ratio was calculated by dividing the SSC value of each juice sample by the percent TA (°Brix ÷ %Acid) [22].

#### 2.3.3. TP and TN Measurements

The TP, which accounts for the majority of phenolics in grape skin extracts, was determined using the Folin–Ciocalteu method [23] with slight modifications. A gallic acid standard curve (*R*^2^ = 0.9997) was used, and the results were described as mg gallic acid equivalents per gram dry weight. The determination of tannins (TN) used the methyl cellulose precipitation method that was proposed by Sarneckis [24]. Moreover, tannins were expressed in mg epicatechin equivalents per g of grape skin powder using a calibration curve (*R*^2^ = 0.9996).

The above measurement processes for TP and TN were performed in triplicate.

### 2.4. Chemometrics and Statistical Analyses

#### 2.4.1. Spectral Clustering

Spectral clustering (SCL) is one of the most attractive statistical techniques for data analysis in many fields [25]. It is a classic graph-based clustering technique that clusters data with a graph partitioning problem, especially using the graph cut method [26]. Specifically, SCL considers each data point as a vertex, measures the edges between two connected data points based on similarity, uses an optimal partition strategy to cut the graph into multiple unconnected subgraphs, and finally divides the data points into different clusters [27]. SCL can converge to the global optimum and is well suited for a sample space of arbitrary shape, especially for a non-convex dataset [28]. In this work, grape samples were grouped into five stages using SCL on the basis of the obtained chemical indicators.

#### 2.4.2. Data Partition

In order to ensure that the distributions of the calibration and prediction sets align with the true distribution, this study employed the sample set partitioning joint x–y distances (SPXY) algorithm [29]. This algorithm selects samples based on the Euclidean distances that combine the spectral space (x) and the target composition space (y) between variables. Initially, the two samples with the greatest Euclidean distances were chosen for the calibration set. Subsequently, the Euclidean distance between each remaining sample and every sample in the calibration set was calculated. The samples with the longest and shortest distances were then added to the calibration set. These steps were repeated until the desired number of samples in the calibration set was reached [30,31]. By utilizing the SPXY algorithm, all grape samples were divided into a calibration set (150 samples) and a prediction set, maintaining a ratio of 3:1.

#### 2.4.3. Preprocessing Transformations

To improve the accuracy of the classification models, spectral preprocessing methods were adopted to transform the spectrum, remove noise due to light scattering, and eliminate baseline drift and other interference information [32]. Therefore, three common preprocessing methods were applied, including the SNV [33], S-G smoothing (window width: 23; polynomial order: 5), and MSC, which were used to deal with the spectral data and helped us improve the robustness of the models.

#### 2.4.4. Variables Selection

The original spectral data consisted of both the relevant samples’ spectral features and background interference [34]. To reduce the variable space effectively, a meticulous selection of wavelengths that carried the most valuable information for prediction was performed [35]. The CARS serves the purpose of eliminating uninformative variables, excluding wavelengths with the strongest correlation coefficients, and enhancing the forecasting accuracy of models [36,37]. Consequently, CARS was adopted for variable selection in this research to establish models quickly and accurately.

The CARS algorithm is based on Darwin’s evolution theory to select the feature variables. The significance of the wavelength variables was determined using the absolute value of the partial least squares (PLS) model. Concretely, it selected N subsets of variables from the Monte Carlo samples in an iterative and competitive manner. Moreover, insignificant wavelength variables were deleted by utilizing the exponential decreasing function and adaptive weighted sampling. Ultimately, the number of selected wavelengths was determined with the subset of the lowest root mean square error of cross-validation (RMSECV) [38,39,40].

#### 2.4.5. Modeling Methods

The intended application and ultimate goals determine the choice of modeling methods. Data matrices were analyzed using the methods of supervised classification, which include objects (input) and corresponding classes (target) [35]. Three different classification models were applied in this work, which included the SVM, 1D-CNN, and SAE. The spectral data were used as input, while the five maturity stages were recorded as targets.

SVM, which is a machine learning method based on statistical learning theory, transforms raw data into a high-dimensional space to construct a hyperplane for classification [41]. According to the principle of minimizing structural risk, the optimal classification hyperplane with a low Vapnik–Chervonenkis dimension is constructed via SVM. As a result, the maximum distance between the classified data and the classification hyperplane occurred [42]. To reduce the computational complexity, the activation function utilizes the RBF kernel in establishing the model. Crucial parameters, including the penalty coefficient (C) and the kernel function parameter (γ), were decided using a randomized search procedure and 5-fold cross-validation, and the ranges of *C* and *γ* were both in the range of 10^−4^ to 10^3^.

**Figure 3:**
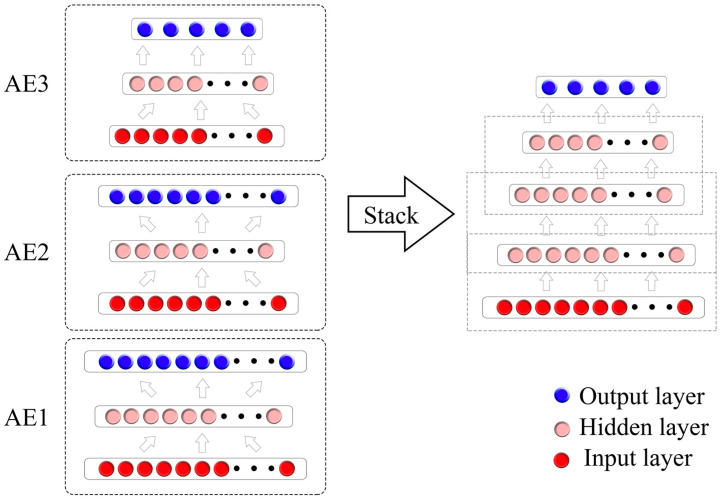
SAE architecture. The input layer is composed of the Vis-NIR spectroscopy data, while the output data represent different ripening stages.

SAE is a deep neural network based on unsupervised learning algorithms consisting of sequentially stacking multiple autoencoders (AE), by which a better classification capability is obtained with the hidden layers added to the simple autoencoders [43]. AE is a neural network model that compresses input data into a low-dimensional code and then reconstructs it back to the original input. The objective of AE is to minimize the reconstruction error, thereby capturing the essential features of the input data. In terms of SAE, each AE’s hidden layer serves as the input layer for the next AE, enabling layer-by-layer training. These hidden input layers are then connected to form the SAE [44]. The process can be seen in Figure 3, and this sequential training approach helps the AE learn higher-level feature representations, thus enhancing the model’s expressive capacity.

The 1D-CNN is a variant of a convolutional neural network that is used to process one-dimensional sequential data that include max dense layers, pooling layers, convolutional layers, and SoftMax layers for one-pixel spectra inputs [35]. Specifically, it extracts local features from the sequence through convolutional operations and reduces feature dimensionality through pooling operations. Nonlinear activation functions introduce nonlinear transformations, while stacked layers and multiple channels enhance the network’s expressive power. Global pooling layers extract fixed-length feature representations, and finally, classification tasks are accomplished through fully connected layers. Table 1 illustrates the parameters and layers of the designed CNN in detail. In our research, the batch size was set to 10, and the learning rate was set to 0.0001. The CNN model was trained for 1000 epochs with a dropout and weight decay method to avoid overfitting.

#### 2.4.6. Model Evaluation

To assess the performance of each supervised classification method, the parameter of classification accuracy was employed. It was evaluated by calculating the percentage of accurately classified grape samples in both the calibration and prediction sets against the total number of samples. In general, the highest accuracy values indicate the optimal classification result in the spectral model.

Within this article, the entire procedure of data analysis and model development was carried out using SPSS 26 (IBM, Armonk, NY, USA) and Python 3.11 on a laptop (Thinkbook 16+, Lenovo Technologies Co., Ltd., Beijing, China).

## 3. Results

### 3.1. Reference Results of the Grape Samples

**Figure 4:**
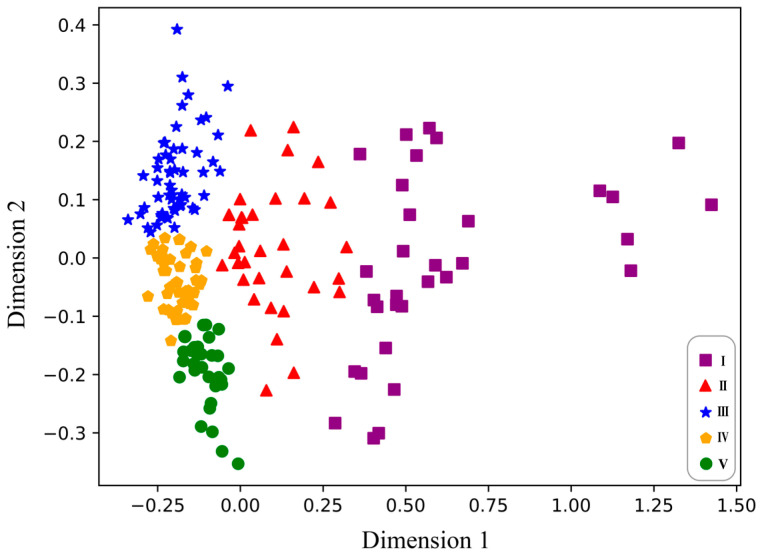
Index clustering divides the ripening stages of grape samples.

The organoleptic properties of wine are largely related to the content of SSC and TA contained in grapes, as well as phenolic compounds extracted from grapes during the winemaking process [45]. Consequently, this study combined the four indices and then divided the grape samples into five maturity stages (I, II, III, IV, and V) using the SCL method. As the number increases, the grape samples become increasingly ripe. There were 32, 34, 55, 49, and 30 grape samples in the I, II, III, IV, and V stages, respectively. The classification diagram is shown in Figure 4. As can be seen, there was a distinct clustering of grape samples in the initial two stages. Additionally, a notable separation was observed between the first two stages and the subsequent three stages, indicating a clear distinction. There were a few scattered samples on the right side of stage I, which might be attributed to the grapes being in the early stages of veraison, the color transformation process of the grapes. These grapes exhibited higher levels of sugar, acid, and phenolic substances [5]. Nevertheless, there existed a certain degree of differentiation among the last three stages, although not as pronounced as in the initial stages. This might have been due to the gradual color changes that occur during the ripening process of the grapes. In a study by Musingarabwi et al. [46], the authors investigated the distinction between grape ripening stages and divided them into five categories: green, early, mid, late, and mature. Similarly, Nogales Bueno et al. [47] successfully differentiated the commencement of grape ripening. However, Martínez Sandoval et al. [48] were unable to identify any discernible distinctions among maturity periods.

Table 2 presents the alteration traits of grapes in terms of SSC, TA, TP, and TN during the five stages of maturity. The SSC values ranged from 11.93% to 18.71%, while the TA values ranged from 3.39 g/L to 12.91 g/L. As anticipated, the SSC exhibited a significant increase (*p* < 0.05) from maturity stage I to V, whereas the TA showed a significant decrease (*p* < 0.05) in the first four stages (I to IV). In addition, there was a slight increase in the latter two stages, IV to V (*p* > 0.05). The obtained results were in line with the previous investigation carried out by Li et al. [49]. This might have been due to the direct accumulation of sugar in the fruit. Following photosynthesis, sugar is transported from the leaves to the fruits primarily via the phloem, and decomposition occurs into soluble sugar in the fruit that is subsequently stored in vacuoles, continuously accumulating during fruit development until full maturity. In addition, the synthesis of organic acids gradually decreases, and the increased cell membrane permeability enables the organic acids in the vacuoles to easily decompose into carbon dioxide and water via respiration, both of which reduce the organic acid levels [50]. As can be seen, as the maturity of the grapes increased, the SSC and TA also changed accordingly. In summary, physiological changes in the SSC and TA contents were closely correlated with grape ripening.

The TP content was between 34.13 and 62.12 mg/g. With the ripening process, the TP content decreased significantly from 62.12 to 34.13 mg/g and subsequently increased to 49.44 mg/g, which was similar to the results of a previous study by Xiao et al. [51].

### 3.2. Spectral Feature

**Figure 5:**
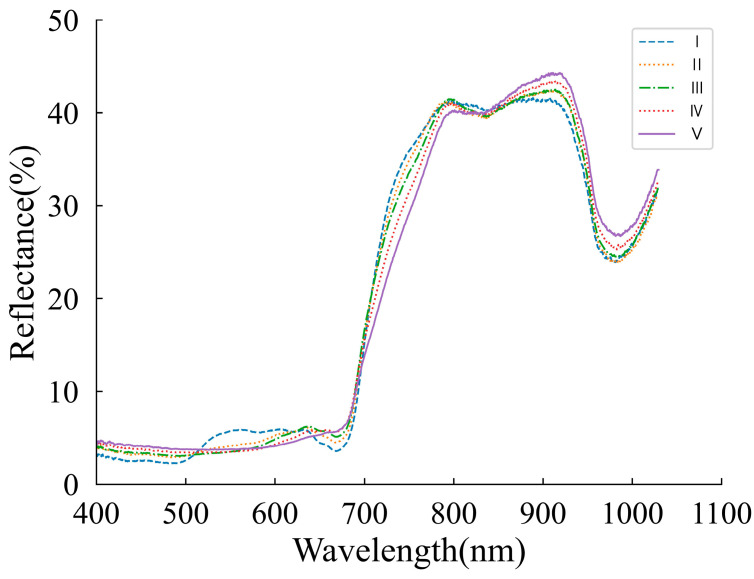
Original Vis-NIR spectral curves during grape veraison until harvest.

Figure 5 represents the diffuse reflectance spectra collected from the different ripening stages of entire grape berries in the wavelength range of 400–1030 nm. In this region, peaks are correlated to the colors that are associated with phenols, carotenoids, anthocyanins, and chlorophyll compounds [52]. Even though there were some differences and crossovers, the trends in the spectra were quite similar. The greatest differences were observed between 400 nm and 780 nm, which might have been caused by discrepancies in pigmented compounds such as chlorophyll and anthocyanin in the grape peels [53,54,55]. Specifically, the variations observed near 535 nm signify discrepancies in the anthocyanin content among the grape berries at different stages of ripeness. Conversely, the discrepancies near 680 nm primarily stem from variations in the chlorophyll content [10]. The five ripening stages were similar in the 780–1030 nm spectral bands. Two small absorption peaks at 820 nm and 970 nm were assigned to the stretching vibration of O-H bonds in sugars and organic and CH_2_ in cellulose, respectively [56].

### 3.3. Discrimination Models on the Full Spectra

**Figure 6:**
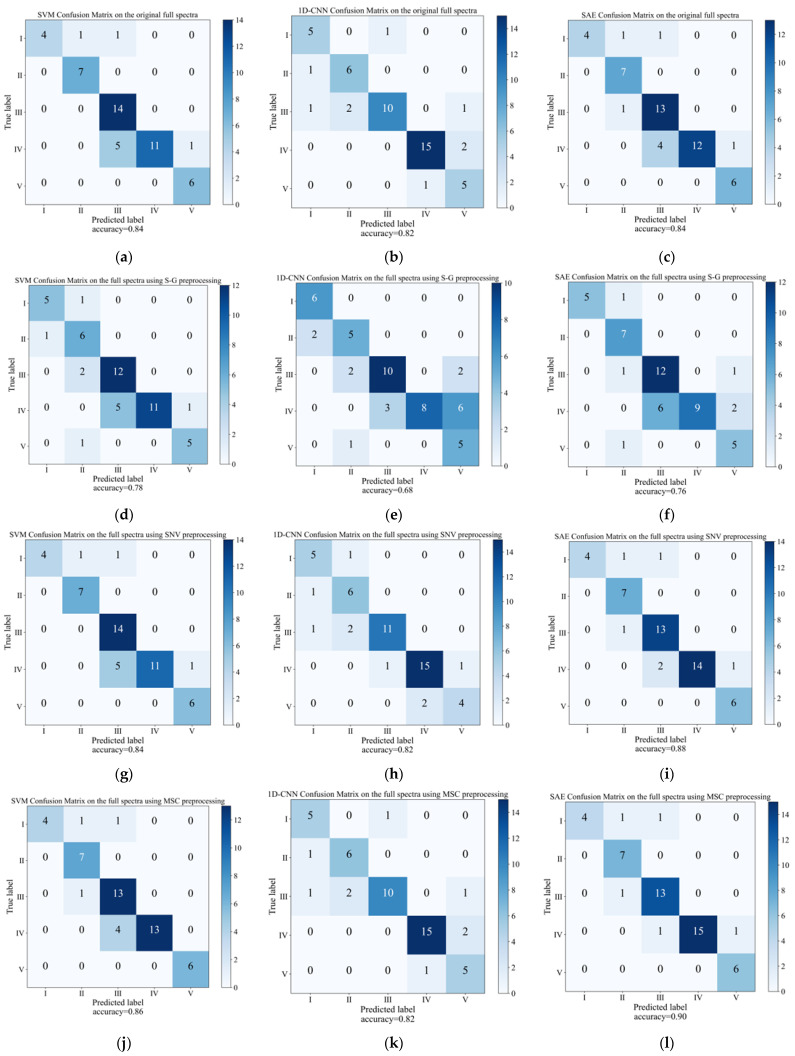
The confusion matrix of prediction sets for SVM, 1D-CNN, and SAE models was established in the full spectra using different preprocessing methods. (**a**–**c**) Original spectra; (**d**–**f**) S-G smoothing; (**g**–**i**) SNV; and (**j**–**l**) MSC.

Three methods of machine learning containing SAE, 1D-CNN, and SVM were employed to construct calibration models on the spectral data that were treated using different preprocessing methods. The relevant parameters of discrimination results can be found in Supplementary Materials Tables S1–S4. Additionally, we have drawn a confusion matrix diagram in Figure 6 to illustrate the discrimination results of the prediction set. As Figure 6 demonstrates, using different spectral preprocessing methods had an impact on the variation in the model results based on the full spectra. In contrast to the preprocessing methods of S-G smoothing, the other preprocessing algorithms, including MSC and SNV, obtained higher accuracies in model discrimination than the original spectral. Following the application of S-G smoothing, the performance of all three models showed a decrease. In comparison to the SVM models, which achieved an accuracy of 78% on the prediction set, the SAE and 1D-CNN models displayed a more substantial decrease in the prediction set accuracy, reaching 76% and 68%, respectively. This could be due to the presence of noise information during the preprocessing step of S-G smoothing [57]. In comparison to the modeling results obtained with SNV spectral preprocessing, the discriminant model exhibited optimal performance when MSC was employed for preprocessing the spectral data. The three models achieved prediction accuracies of 88%, 82%, and 90%, respectively. Therefore, the MSC proved to be the most appropriate preprocessing technique for the discriminant models, effectively capturing the variations across different ripening stages when applied to the complete spectra.

With respect to the various stages, the classification performance of the discriminant models exhibited variation, with the ripening stage II consistently achieving the highest level of classification accuracy. However, stage III and stage IV were found to be easily misclassified. This indicated that different stages of grape ripening could influence the discrimination results. This may have occurred because the physical and chemical properties of grape berries during ripening changed dramatically after the pre-veraison period. Here, the grape berries begin to soften, change color, and increase in size and sugar content [51]. Furthermore, there may have been minor changes from the later stage of the veraison to the completion of the veraison. Among all three discriminative models, the 1D-CNN model exhibits the lowest impressive outcomes, exhibiting an accuracy of less than 85% for the prediction set. This can be attributed to the limited sample size, which restricts the model’s complexity and generalization capability [58]. In contrast, the SAE model performs the best, obtaining classification accuracies of 90% for the prediction sets and 100% for the calibration sets. This is likely because the SAE model leverages the reconstruction ability of the autoencoder to generate composite samples, thereby expanding the training data size, improving data quality, mitigating overfitting issues, and enhancing model accuracy. Therefore, the SAE discriminant models of the ripening stages based on the full spectra yielded satisfactory outcomes when applied to the complete spectra with the appropriate spectral preprocessing techniques.

### 3.4. Effective Wavelength Selection

The original spectral data encompassed a total of 1507 distinct wavelengths. The reflectance spectrum of the experimentally acquired material contained more bands, and there were large correlations and redundant information between the bands. In order to improve the detection accuracy and detection speed of the model, feature wavelength extraction from the raw spectrum was required. Therefore, CARS was employed to identify the optimal wavelengths.

**Figure 7:**
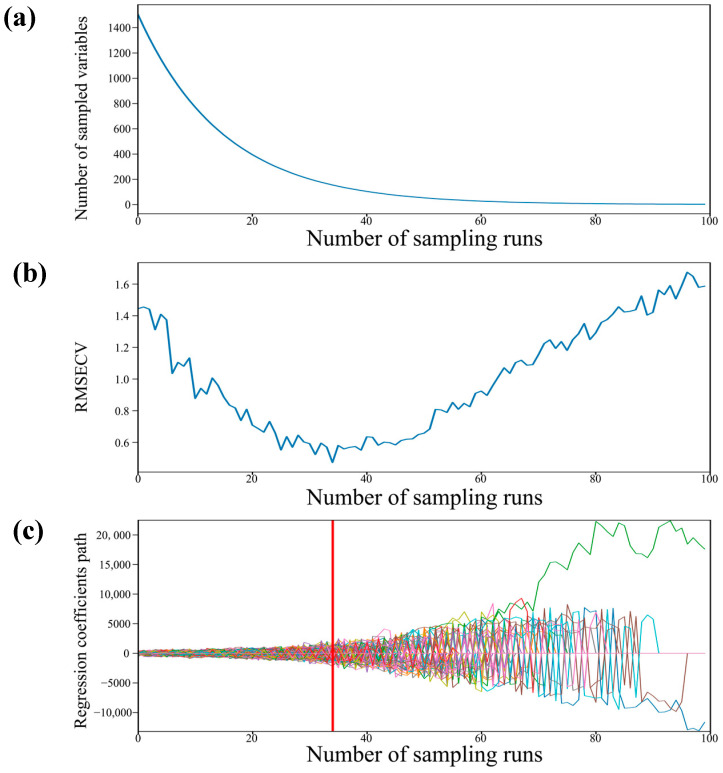
Effective spectral data selection based on the CARS, (**a**) the number of sampled variables, (**b**) 10-fold RMSECV values, and (**c**) the trajectory of the regression coefficient for each variable as the number of sampling runs increased.

**Figure 8:**
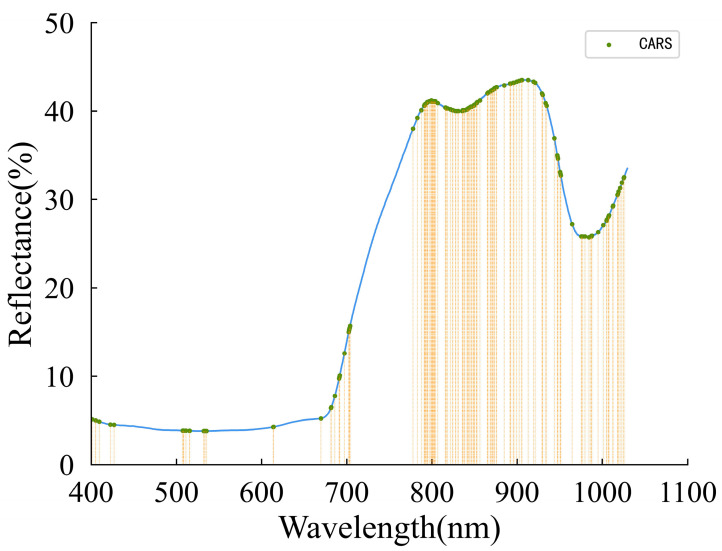
The location of the variables selected with the CARS for ripening stage detection.

The CARS variable selection procedure for the grape samples based on the complete spectral data is shown in Figure 7. The 100 Monte Carlo sampling runs were followed by a 10-fold cross-validation to determine the variables’ significance [59]. Moreover, the minimum RMSECV corresponding to the PLS model established by the 100 sampling runs was taken as the optimal result. As depicted in Figure 7a, a steep decline is observed initially, followed by a relatively stable number of sampled bands as the number of sampling runs increases. This highlighted the efficient and swift selection process of the CARS algorithm. The dynamic trend of the 10-fold RMSECV values was displayed in Figure 7b as the number of sampling runs gradually increased. At first, the RMSECV values exhibited a gradual decline as the number of sampling runs increased, possibly attributed to the stepwise elimination of uninformative variables. Subsequently, as the valuable spectral variables were progressively eliminated, the RMSECV values started to rise. Finally, it can be seen that the regression coefficients of each variable are located at the position of the vertical straight line in Figure 7c when the RMSECV value reaches the minimum value, at which time the sampling runs were conducted 34 times. Consequently, having utilized the CARS method, a total of 154 effective wavelengths were chosen from the complete spectra, leading to a reduction of over 89% in data volume. Moreover, the distribution of the retained wavelengths is displayed in Figure 8. Apparently, the effective spectra were predominantly concentrated around the peaks and valleys of the waveforms. This observation indicates that the selected wavelengths contain valuable information pertaining to sugars, acids, and phenolic substances.

### 3.5. Discrimination Models on the Selected Spectra

**Figure 9:**
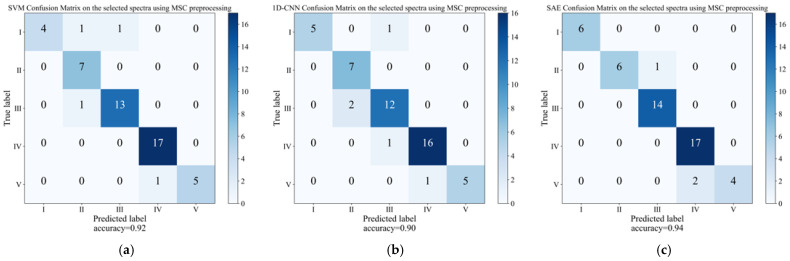
The confusion matrix of prediction sets for SVM, 1D-CNN, and SAE models was established in the selected spectra using MSC preprocessing methods. (**a**) SVM, (**b**) 1D-CNN, and (**c**) SAE.

By employing the CARS method to discern the crucial spectral region, the quantity of input variables can be minimized, thereby facilitating the construction of more stable and streamlined models. The relevant parameters of discrimination results can be found in Supplementary Material Table S5. Figure 9 shows the influence of the SVM, 1D-CNN, and SAE discriminant models on the ripening stages of the samples in the prediction set, and they were constructed after the extraction of feature wavelengths from the raw spectra using the CARS method. It is evident that the accuracy of the models established using the characteristic wavelengths filtered surpasses that of the models constructed using the entire spectrum of wavelengths, yielding, in all cases, calibration accuracies that were not less than 94% and prediction accuracies that were greater than or equal to 90%. Among them, the SAE model achieves the highest discrimination accuracy in the prediction set, reaching up to 94%. Furthermore, chi-squared tests or Fisher’s exact tests were conducted on the accuracy and full band accuracy of the predicted set for each stage after the feature wavelength screening. The results are summarized in Table 3. The table reveals that there was a significant improvement in the accuracy of the grape sample prediction established in stage IV (*p* < 0.05). The accuracy of the judgment in stages I and III showed improvement, although it was not statistically significant (*p* > 0.05). The judgment effect in stage II remained unchanged, while in stage V, there was a slight decrease in the judgment effect, which was also not statistically significant (*p* > 0.05). Interestingly, there was a higher tendency for misjudgment at stage IV. This could have been attributed to the fact that the selected characteristic wavelength retained a greater amount of spectral information pertaining to the TA content. In stage V, the grape samples have already reached physiological maturity, and the TA content has stabilized, making it more challenging to differentiate between samples at this stage [60].

Among the analyzed models, 1D-CNN was found to be the most sensitive to wavelength reduction. The accuracy of the calibration set decreased from 98% to 94%, while the accuracy of the prediction set increased from 82% to 90%. Based on the selected spectral wavelengths, the SAE model emerged as the most successful among all of the discriminant models, whereas the accuracy of the prediction reached 94%. In general, running the simplified SAE model with selected wavelengths significantly reduced the dimension of the input variables, thus significantly reducing the computational complexity. Therefore, in this study, the spectra were preprocessed with MSC, and the characteristic wavelengths were selected using the CARS method. An SAE approach was then used to model the selected wavelengths, resulting in the development of a high-precision discrimination model for grape ripening stages.

## 4. Discussion

Several researchers have investigated the viability of using Vis-NIR for identifying fruit ripening stages and predicting their composition. Nevertheless, it is worth noting that the manual division of grape maturity stages based on time or a principal component analysis of the spectra, along with the use of traditional machine learning methods for model establishment, have been the common practices used in many studies [10,19,51]. However, vineyards possess inherent heterogeneity in both temporal and spatial dimensions, resulting in variations in vineyard maturity even during the same period. Furthermore, sugars, acids, and phenolic compounds are crucial for the quality, color stability, mouthfeel, and flavor of red wine, all of which are closely related to its maturity [61]. Therefore, a discriminative method that combines these indicators to assess maturity, eliminating the need for individual quantitative predictions, is considered highly valuable. By doing so, it reduces the number and complexity of models used, resulting in a more streamlined and insightful approach. Therefore, the present article adopted a spectral clustering method that considered multiple grape indicators, aiming to discriminate between the different stages of grape maturity more effectively. By leveraging a comprehensive range of grape indicators, this approach sought to precisely identify the corresponding indicators for each stage. This method aids in the efficient and selective harvesting of grapes to achieve the desired grape maturity according to winemaking requirements, thereby ensuring optimal quality standards and enhancing the style and quality of the wine. Besides the effectiveness of traditional supervised classification methods coupled with manual selection of characteristic spectral regions in identifying sample features, we investigated the viability of employing deep learning techniques to distinguish grape ripening stages and assessed its efficacy in comparison with subsequent predictions based on CARS-selected spectral wavelengths. In this study, we opted for two deep neural network learning methods, namely SAE and 1D-CNN. These methods possess the ability to autonomously learn and extract features by leveraging multiple convolutional layers, making them well suited for classification tasks. It is worth noting that in previous spectroscopic studies, deep learning methods have consistently showcased superior performance when compared to traditional machine learning approaches [62,63]. Regarding the application of deep learning in near-infrared NIR spectroscopy, Basile et al. undertook an endeavor to predict the texture parameters and the SSC in pristine berries [64]. The deep artificial neural network (ANN) models exhibited superior performance compared to the PLS models, especially after removing noninformative spectral ranges for samples. Likewise, in our research, the SAE model, utilizing feature spectra filtered via CARS coupled with MSC preprocessing, exhibited preferable performance compared to other discriminative models. It achieved remarkable classification accuracies of 100% and 94% for the calibration and prediction sets, respectively, surpassing the model constructed solely using the entire spectra. Such advancements enable the precise identification of grape ripening stages within a concise timeframe. Nevertheless, as novel samples emerge, continuous updates are imperative to enhance the efficacy of the detection system. In this context, the SAE model demonstrates remarkable adaptability in extracting intricate spectral features from raw data, showcasing its superior self-learning capacity. This characteristic renders it highly suitable for the development of real-time monitoring systems, catering to the demands of contemporary industries for automated detection.

Considering the limitations of this study, it should be noted that although feature wavelength selection was performed, a relatively large number of wavelengths were chosen without effectively minimizing redundancy. In future research, improving modeling speed can be achieved by optimizing the feature wavelength selection method to precisely identify the wavelengths that are specifically correlated with the target indicators. Moreover, both the SAE and 1D-CNN models were designed with a reduced number of structural layers. This decision was influenced by the fact that the spectral data provided only one-dimensional information, and utilizing excessively deep networks could potentially result in overfitting. It is important to note that deep learning models with higher depth are generally more suitable for larger datasets. Moving forward, expanding the collection of grape samples from diverse sources and incorporating samples from different years will enable us to refine the model. Additionally, implementing fine-tuning and training using samples collected over multiple years will enhance its stability and universality. These efforts possess the potential to expand the layers of the SAE and 1D-CNN models, establishing robust models and improving the accuracy of classification, thus ensuring ongoing advancements in this field.

## 5. Conclusions

In this study, we aimed to investigate whether integrating Vis-NIR technology with stoichiometric approaches that include feature selection and supervised classification could serve as an innovative technique for identifying grape ripening stages. In this study, we succeeded in extracting spectral features from grape samples using CARS functional wavelength filtering techniques to reduce the computational time and improve the detection capabilities of the model. The SAE model, which autonomously learns deep features from feature spectra, showed commendable performance during our experiments. The calibration set achieved 100% accuracy, while the prediction set achieved 94% accuracy. In addition, we conducted measurements to evaluate the quality characteristics of the grapes at each stage. By synergistically leveraging the advantages of rapid data collection through Vis-NIR technology and the self-learning capabilities of the SAE model, we can develop an online detection system to accurately determine the ripening stages of grapes. With no need for complex sample processing or chemical reagents, Vis-NIR technology enables the rapid acquisition of spectra containing valuable elemental information, while deep learning techniques excel in interpreting spectral data. Our study provides fundamental implications for future applications in wine production and the development of optoelectronic instruments tailored to the specific needs of the wine industry. Nevertheless, further research is required to improve the accuracy and robustness of the prediction models, which requires the use of a significant number of grape samples that include greater varietal diversity. In addition, it is important to recalibrate the grape prediction models for different crop years to improve their reliability and practicality.

## Figures and Tables

**Table 1:** Layers and parameters of the one-dimensional convolutional neural network architecture.

Layers	Parameters	Activation	Additional Processing
Convolution-1D(1)	Kernel number = 64	Relu	Batch normalization
	Kernel size = 2, strides = 2		
Maxpooling	Size = 3, Strides = 1	--	--
Convolution-1D(2)	Kernel number = 32	Relu	Batch normalization
	Kernel size = 2, strides = 2		
Dense(1)	Neurons = 512(128)	--	Batch normalization
			Drop out = 0.5
Dense(2)	Neurons = 64(32)	--	Batch normalization
			Drop out = 0.5
Dense(3)	Neurons = 5	--	--

Notes: The numbers in parentheses correspond to the parameters used for modeling the selected characteristic wavelength spectral data.

**Table 2:** SSC, TA, TP, and TN of the grape samples during different ripening stages.

Ripening Stage	SSC (°Brix)	TA (g/L)	SSC/TA	TP (mg/g)	TN (mg/g)
I	11.93 ± 3.07 e	12.91 ± 4.49 a	0.92 ± 0.59 d	62.12 ± 8.57 a	48.60 ± 11.17 a
II	14.84 ± 1.24 d	5.92 ± 1.55 b	2.51 ± 0.77 c	45.79 ± 5.136 c	30.64 ± 5.14 b
III	15.71 ± 1.27 c	3.76 ± 0.79 c	4.18 ± 1.01 b	34.13 ± 3.08 e	14.96 ± 3.53 e
IV	17.78 ± 0.65 b	3.39 ± 0.35 d	5.25 ± 0.56 a	40.86 ± 2.93 d	19.12 ± 3.21 d
V	18.71 ± 0.71 a	3.42 ± 0.38 d	5.47 ± 0.66 a	49.44 ± 3.65 b	25.49 ± 3.16 c

Notes: The lowercase letters indicate significant differences between the maturity stages within the same indicator (*p* < 0.05).

**Table 3:** Chi-squared and Fisher’s exact tests for the prediction results of the SVM, SAE, and 1D-CNN models on the selected spectra.

Stages	MSC		MSC-CARS		*p*-Value
	T	F	T	F	
I	13	5	15	3	0.691 (Fisher)
II	20	1	20	1	1
III	36	6	39	3	0.48
IV	43	8	50	1	0.036
V	17	1	14	4	0.338 (Fisher)

Notes: The parentheses indicate the use of Fisher’s exact tests, while the others use Chi-squared tests.

## Data Availability

The related data and methods are presented in this paper. Additional inquiries should be addressed to the corresponding author.

## Supplementary Materials

The following supporting information can be downloaded at https://www.mdpi.com/article/10.3390/foods12234371/s1, Table S1: Discriminant results of the SVM, SAE, and 1D-CNN models on the raw spectra; Table S2: Discriminant results of the SVM, SAE, and 1D-CNN models on the S-G smoothing preprocessing; Table S3: Discriminant results of the SVM, SAE, and 1D-CNN models on the SNV preprocessing; Table S4: Discriminant results of the SVM, SAE, and 1D-CNN models on the MSC preprocessing; Table S5: Discriminant results of the SVM, SAE, and 1D-CNN models on the MSC-CARS preprocessing.
