# Supplementary material for: Discrimination of Maturity Stages of Cabernet Sauvignon Wine Grapes Using Visible–Near-Infrared Spectroscopy
Source: Foods. 2023 Dec 4;12(23):4371. doi: 10.3390/foods12234371 (PMC10706061; doi:10.3390/foods12234371)
Supplement: Supplementary file 1 [file foods-12-04371-s001.zip › foods-2700798-supplementary.pdf]

TP: true positive, the number of correctly recognized class.

TN: true negatives, the number of correctly recognized examples that do not belong to the class.

FP: false positives, examples that either were incorrectly assigned to the class

FN: false negatives, that were not recognized as class examples.

Precision: the number of correctly classified positive examples divided by the number of examples labeled by the system as positive.

$$Precision = \frac{TP}{TP + FP}$$

Recall: the number of correctly classified positive examples divided by the number of positive examples in the data.

$$Rcall = \frac{TP}{TP + FN}$$

F1-score: a combination of the above.

$$F1 - score = \frac{2 * Precision * Recall}{Precision + Recall}$$

Accuracy: the average per-class effectiveness of a classifier.

$$Accuracy = \frac{TP + TN}{TP + TN + FP + FN}$$

Error rate: the average per-class classification error.

$$Error\ rate=1\text{-}Accuracy$$

Tabel S1. Discriminant results of the SVM, SAE, and 1D-CNN models on the raw spectra.

RAW							
models	stage	Calibration set			Prediction set		
		precision	recall	f1-score	precision	recall	f1-score
SVM	1	1.00	1.00	1.00	1.00	0.67	0.80
	2	1.00	1.00	1.00	0.88	1.00	0.93
	3	1.00	1.00	1.00	0.70	1.00	0.82
	4	1.00	1.00	1.00	1.00	0.65	0.79
	5	1.00	1.00	1.00	0.86	1.00	0.92
	accuracy		1.00			0.84	
	error rate		0.00			0.16	
1D-CNN	1	1.00	1.00	1.00	0.71	0.83	0.77

	2	1.00	1.00	1.00	0.75	0.86	0.80
	3	1.00	1.00	1.00	0.91	0.71	0.80
	4	0.97	0.94	0.95	0.94	0.88	0.91
	5	0.92	0.96	0.94	0.63	0.83	0.71
	accuracy		0.98			0.82	
	error rate		0.02			0.18	
SAE	1	1.00	1.00	1.00	1.00	0.67	0.80
	2	1.00	1.00	1.00	0.78	1.00	0.88
	3	1.00	1.00	1.00	0.72	0.93	0.81
	4	1.00	1.00	1.00	1.00	0.71	0.83
	5	1.00	1.00	1.00	0.86	1.00	0.92
	accuracy		1.00			0.84	
	error rate		0.00			0.16	

Tabel S2. Discriminant results of the SVM, SAE, and 1D-CNN models on the S-G smoothing preprocessing.

S-G smoothing							
models	stage	Calibration set			Prediction set		
		precision	recall	f1-score	precision	recall	f1-score
SVM	1	1.00	1.00	1.00	0.83	0.83	0.83
	2	1.00	1.00	1.00	0.60	0.86	0.71
	3	1.00	1.00	1.00	0.71	0.86	0.77
	4	0.97	0.97	0.97	1.00	0.65	0.79
	5	0.96	0.96	0.96	0.83	0.83	0.83
	accuracy		0.99			0.78	
	error rate		0.01			0.22	
1D-CNN	1	1.00	0.96	0.98	0.75	1.00	0.86
	2	1.00	0.85	0.92	0.63	0.71	0.67
	3	0.89	0.95	0.92	0.77	0.71	0.74
	4	0.90	0.88	0.89	1.00	0.47	0.64
	5	0.85	0.96	0.90	0.38	0.83	0.53
	accuracy		0.91			0.68	
	error rate		0.09			0.32	
SAE	1	1.00	1.00	1.00	1.00	0.83	0.91
	2	1.00	1.00	1.00	0.70	1.00	0.82
	3	1.00	1.00	1.00	0.67	0.86	0.75
	4	1.00	1.00	1.00	1.00	0.53	0.69
	5	1.00	1.00	1.00	0.63	0.83	0.71
	accuracy		1.00			0.76	
	error rate		0.00			0.24	

Tabel S3. Discriminant results of the SVM, SAE, and 1D-CNN models on the SNV preprocessing.

SNV							
models	stage	Calibration set			Prediction set		
		precision	recall	f1-score	precision	recall	f1-score
SVM	1	1.00	1.00	1.00	1.00	0.67	0.80
	2	1.00	1.00	1.00	0.88	1.00	0.83
	3	1.00	1.00	1.00	0.70	1.00	0.82
	4	1.00	1.00	1.00	1.00	0.65	0.79
	5	1.00	1.00	1.00	0.86	1.00	0.92
	accuracy		1.00			0.88	
	error rate		0.00			0.12	
1D-CNN	1	1.00	1.00	1.00	0.71	0.83	0.77
	2	0.96	0.96	0.96	0.67	0.86	0.75
	3	1.00	1.00	1.00	0.92	0.79	0.85
	4	0.94	0.94	0.94	0.88	0.88	0.88
	5	0.96	0.96	0.96	0.80	0.67	0.73
	accuracy		0.98			0.82	
	error rate		0.02			0.18	
SAE	1	1.00	1.00	1.00	1.00	0.67	0.80
	2	1.00	1.00	1.00	0.78	1.00	0.88
	3	1.00	1.00	1.00	0.81	0.93	0.87
	4	1.00	1.00	1.00	1.00	0.82	0.90
	5	1.00	1.00	1.00	0.86	1.00	0.92
	accuracy		1.00			0.88	
	error rate		0.00			0.12	

Tabel S4. Discriminant results of the SVM, SAE, and 1D-CNN models on the MSC preprocessing.

MSC							
models	stage	Calibration set			Prediction set		
		precision	recall	f1-score	precision	recall	f1-score
SVM	1	1.00	1.00	1.00	1.00	0.67	0.80
	2	1.00	1.00	1.00	0.78	1.00	0.88
	3	1.00	1.00	1.00	0.72	0.93	0.81
	4	1.00	1.00	1.00	1.00	0.76	0.87
	5	1.00	1.00	1.00	1.00	1.00	1.00
	accuracy		1.00			0.86	
	error rate		0.00			0.14	
1D-CNN	1	1.00	1.00	1.00	0.71	0.83	0.77
	2	1.00	1.00	1.00	0.71	0.86	0.80
	3	1.00	1.00	1.00	0.91	0.71	0.80

	4	0.97	0.94	0.95	0.94	0.88	0.91
	5	0.92	0.96	0.94	0.63	0.83	0.71
	accuracy		0.98			0.82	
	error rate		0.02			0.18	
SAE	1	1.00	1.00	1.00	1.00	0.67	0.80
	2	1.00	1.00	1.00	0.78	1.00	0.88
	3	1.00	1.00	1.00	0.87	0.93	0.90
	4	1.00	1.00	1.00	1.00	0.88	0.94
	5	1.00	1.00	1.00	0.86	1.00	0.92
	accuracy		1.00			0.90	
	error rate		0.00			0.10	

Tabel S5. Discriminant results of the SVM, SAE, and 1D-CNN models on the MSC-CARS preprocessing.

MSC-CARS							
models	stage	Calibration set			Prediction set		
		precision	recall	f1-score	precision	recall	f1-score
SVM	1	1.00	1.00	1.00	1.00	0.67	0.80
	2	1.00	1.00	1.00	0.78	1.00	0.88
	3	1.00	1.00	1.00	0.93	0.93	0.93
	4	1.00	1.00	1.00	0.94	1.00	0.97
	5	1.00	1.00	1.00	1.00	0.83	0.91
	accuracy		1.00			0.92	
	error rate		0.00			0.08	
1D-CNN	1	1.00	1.00	1.00	1.00	0.83	0.91
	2	1.00	1.00	1.00	0.70	1.00	0.82
	3	1.00	1.00	1.00	0.86	0.86	0.86
	4	0.97	0.94	0.95	1.00	0.94	0.97
	5	0.92	0.96	0.94	1.00	0.83	0.91
	accuracy		0.94			0.90	
	error rate		0.06			0.10	
SAE	1	1.00	1.00	1.00	1.00	1.00	1.00
	2	1.00	1.00	1.00	1.00	0.86	0.92
	3	1.00	1.00	1.00	0.93	1.00	0.97
	4	1.00	1.00	1.00	0.89	1.00	0.94
	5	1.00	1.00	1.00	1.00	0.67	0.80
	accuracy		1.00			0.94	
	error rate		0.00			0.06	
